# Notes and News

**Published:** 1893-03-04

**Authors:** 


### Notes and News.

Collected and Communicated.

The editorial and publishing offices of The Hospital have been removed to more extensive premises, comprising the whole of the three upper floors at 428, Strand, W.C. This removal has been rendered unavoidable owing to the expiration of the lease of premises at 140, Strand, and is further due to the necessity which has arisen for securing increased accommodation for the growing demands of the business.

In Vienna, at Professor Stricker's Institute of Experimental Pathology, some of the medical men have experimented on themselves with Koch's cholera bacilli. The bacilli used were of two kinds, some purely cultivated, and others cultivated in gelatine. Doses under one cubic centimeter made no apparent change in the health of the subject. Doses of one cubic centimeter and 1½ cubic centimeter produced diarrhœa and a rise of temperature, but patients were restored to health within a few days.

The annual meeting of the Charity Organisation Society was held at the Mansion House last week, the Lord Mayor presiding. Mr. C. G. Roundell, in his speech, gave promise to an awakening energy and helpfulness on the part of the Society. The scope for this appears to be opened by the pending alteration in the poor laws, which will afford the C.O.S. opportunities of cultivating a friendly spirit alongside of the judicial character of its investigations. If this is carried into effect, the impression which the Society has succeeded in giving, that it regards all as criminals until proved otherwise, will be effectually removed, and the tone of future meetings will justly assume a less apologetic tone than was apparent on the present occasion.

At the quarterly general court of the Middlesex Hospital, held last week, Lord Sandhurst, chairman of the Weekly Board, in moving the adoption of the report and speaking on the subject of hospital finances, said that he was of the opinion that money given by the public was not intended to be hoarded, but to be used discriminately, leaving posterity to care for their own sick and poor. This opinion is by no means held generally. Many institutions would receive such principles as revolutionary, even whilst suffering from lack of funds for immediate use. Nevertheless, many hospitals have yearly to encroach on invested funds, which, whilst they last, give an unwise sense of security which may tend to diminish the energy of the management in collecting for the institution.

We learn that the new wing at the Hospital for Sick Children, Great Ormond Street, is to be opened by the Prince and Princess of Wales in June. As at the recent ceremony at St. Mary's Hospital, purses of £5 5s. are to be presented towards removing the building debt of £12,000. The purses are to be presented to the Princess by children, nominated by any collector of a sum not less than that mentioned above.

A pleasant entertainment was given last week to the patients of the Brompton Hospital, under the direction of Mrs. Gouraud, of Upper Norwood. Sullivan's "Trial by Jury," was admirably performed by an amateur company, many of whom were connected with medical institutions. Both patients and guests expressed themselves delighted by the result of the kind efforts made in their behalf.

Mr. Francis Brocklehurst has just given 13 acres of land to the Macclesfield Town Council for the purpose of laying out a pleasure and recreation ground on the east side of the town. The cost of erecting a caretaker's house and making the necessary approaches is borne by the generous patron, which work it is estimated will cost him £5,000. Mr. Brocklehurst has put one condition, that is, that the maintenance of the ground and of the building will be defrayed by the Town Council, who gratefully accept the offer.

Oxford University is to have its own fever hospital. This decision has been arrived at owing to the closing of the fever wards at the Radcliffe Infirmary. The proposed site is to be near the city fever hospital at Cold Harbour, and the whole cost of establishing the hospital is estimated at £4,500. The maintenance, not including direct cost of patients, to be covered by fees, is calculated at £150. The total cost to the University is expected to amount to £420 per annum, and to provide for this sum it is proposed to tax all members of the University one shilling per quarter, to be regarded amongst University dues. The Sarah Acland Nursing Home Committee have undertaken the administration of the new hospital, which agreement is to be terminable at the expiration of five years if so desired by University or Committee.

The proposal to establish a floating cholera or infectious diseases hospital in the Thames is assuming definite proportions. The position most favoured is between the Blackfriars and London Bridges. The gangway would be less than 30 feet from the shore, the vessel a flat-bottomed one, would be 100 feet long and 25 feet wide, and would be constructed to contain 50 patients. The Sanitary Committee and City Commissioners of Sewers are putting forward the matter, and the Thames Conservancy have stated in Parliament that they will carefully guard the interests of citizens in the matter of infection from the hospital. With cholera already at Marseilles, no time should be lost in effective preparations in case of a return of the epidemic with the spring. In America the matter is receiving the closest attention, and England, in nearer proximity to infected localities, cannot afford to ignore the threatened danger.

